# Visceral Adiposity in Relation to Body Adiposity and Nutritional Status in Elderly Patients with Stable Coronary Artery Disease

**DOI:** 10.3390/nu13072351

**Published:** 2021-07-09

**Authors:** Bartosz Hudzik, Justyna Nowak, Janusz Szkodziński, Barbara Zubelewicz-Szkodzińska

**Affiliations:** 1 Department of Cardiovascular Disease Prevention, Department of Metabolic Disease Prevention, Faculty of Health Sciences, Medical University of Silesia, 41-902 Bytom, Poland; justyna.nowak@sum.edu.pl; 2 Third Department of Cardiology, Silesian Center for Heart Disease, Faculty of Medical Sciences, Medical University of Silesia, 41-800 Zabrze, Poland; janszkod@poczta.onet.pl; 3 Department of Nutrition-Related Disease Prevention, Department of Metabolic Disease Prevention, Faculty of Health Sciences, Medical University of Silesia, 41-902 Bytom, Poland; basiazub@poczta.onet.pl; 4 Department of Endocrinology, District Hospital, 41-940 Piekary Śląskie, Poland

**Keywords:** visceral adiposity, obesity, nutritional status, coronary artery disease, VAI, BMI, CONUT score

## Abstract

**Introduction**: The accumulation of visceral abdominal tissue (VAT) seems to be a hallmark feature of abdominal obesity and substantially contributes to metabolic abnormalities. There are numerous factors that make the body-mass index (BMI) a suboptimal measure of adiposity. The visceral adiposity index (VAI) may be considered a simple surrogate marker of visceral adipose tissue dysfunction. However, the evidence comparing general to visceral adiposity in CAD is scarce. Therefore, we have set out to investigate visceral adiposity in relation to general adiposity in patients with stable CAD. **Material and methods**: A total of 204 patients with stable CAD hospitalized in the Department of Medicine and the Department of Geriatrics entered the study. Based on the VAI-defined adipose tissue dysfunction (ATD) types, the study population (N = 204) was divided into four groups: (1) no ATD (N = 66), (2) mild ATD (N = 50), (3) moderate ATD (N = 48), and (4) severe ATD (N = 40). Nutritional status was assessed using the Controlling Nutritional Status (CONUT) score. **Results**: Patients with moderate and severe ATD were the youngest (median 67 years), yet their metabolic age was the oldest (median 80 and 84 years, respectively). CONUT scores were similar across all four study groups. The VAI had only a modest positive correlation with BMI (r = 0.59 *p* < 0.01) and body adiposity index (BAI) (r = 0.40 *p* < 0.01). There was no correlation between VAI and CONUT scores. There was high variability in the distribution of BMI-defined weight categories across all four types of ATD. A total of 75% of patients with normal nutritional status had some form of ATD, and one-third of patients with moderate or severe malnutrition did not have any ATD (*p* = 0.008). In contrast, 55–60% of patients with mild, moderate, or severe ATD had normal nutritional status (*p* = 0.008). ROC analysis demonstrated that BMI and BAI have poor predictive value in determining no ATD. Both BMI (AUC 0.78 *p* < 0.0001) and BAI (AUC 0.66 *p* = 0.003) had strong predictive value for determining severe ATD (the difference between AUC 0.12 being *p* = 0.0002). However, BMI predicted mild ATD and severe ATD better than BAI. **Conclusions**: ATD and malnutrition were common in patients with CAD. Notably, this study has shown a high rate of misclassification of visceral ATD via BMI and BAI. In addition, we demonstrated that the majority of patients with normal nutritional status had some form of ATD and as much as one-third of patients with moderate or severe malnutrition did not have any ATD. These findings have important clinical ramifications for everyday practice regarding the line between health and disease in the context of malnutrition in terms of body composition and visceral ATD, which are significant for developing an accurate definition of the standards for the intensity of clinical interventions.

## 1. Introduction

Obesity has become a worldwide pandemic. It is linked to numerous conditions including, but not limited to, cardiovascular disease (CVD), prediabetes and type 2 diabetes mellitus, hypertension, hyperlipidemia, sleep apnea, and a few types of certain malignancies [1]. Adipose tissue has long been viewed as a storage place for fatty acids. However, this traditional view has been replaced by the theory that adipose tissue is a crucial organ of the endocrine system, which releases various adipokines connected to the pathogenesis of a myriad of obesity-related metabolic disturbances. Obesity is an established risk factor for atherosclerosis and coronary artery disease (CAD) and can accelerate both conditions via a wide variety of actions, such as a rise in blood pressure and glucose concentration, abnormal lipid profiles, and most significantly, by creating a systemic inflammatory milieu. More importantly, a growing body of evidence suggests that many adipokines may directly mediate atherogenesis by affecting the function of endothelial cells, arterial smooth muscle cells, and macrophages within arterial walls [2,3]. The accumulation of visceral abdominal tissue (VAT) seems to be a hallmark feature of abdominal obesity and substantially contributes to metabolic abnormalities [4]. In that respect, visceral adiposity may explain more of the variance in the CVD risk factors than would general obesity [5].

Numerous modalities have been used to evaluate body composition. Dual-energy X-ray absorptiometry (DXA) allows for the fast and noninvasive assessment of fat mass (FM) and fat-free mass (FFM) and is considered to be the gold standard in clinical research. Notwithstanding, the need for specialized radiology equipment and the high costs of the method are the major shortcomings of DXA [6]. Conversely, anthropometric techniques are considered simple, rapid, and inexpensive and are readily available and feasible in everyday clinical practice. These include general and visceral obesity anthropometric measures such as the body-mass index (BMI), waist circumference (WC), hip circumference (HC), waist-to-hip ratio (WHR), waist-to-height ratio (WHtR), and body adiposity index (BAI).

BMI is the most commonly used method to measure body fat [7]. There are numerous factors that make the BMI a suboptimal measure of adiposity. First and foremost, it is hardly a surrogate measure of adiposity because it measures excess weight rather than excess fat. In addition, BMI does not account for the distribution of body fat [8].

The BAI was proposed to overcome the limitations of the BMI in discriminating between fat and lean body mass. It is calculated from hip circumference and height. The final formula was shown to predict the percentage of body adiposity measured by DXA [9,10,11].

The visceral adiposity index (VAI) is a gender-specific model that is calculated by using simple anthropometric parameters (BMI and WC) and biochemical parameters (high-density lipoprotein (HDL) cholesterol and triglyceride levels). Therefore, the VAI formula is indicative of fat distribution and function. VAI may be considered a simple surrogate marker of visceral adipose tissue dysfunction (VAD) and demonstrates a strong link to VAT measured with magnetic resonance imaging (MRI). More importantly, VAT-associated cardiometabolic risk was demonstrated to predict both cardiovascular and cerebrovascular events [12,13,14].

More recent attention has also focused on the effect of nutritional status on cardiovascular outcomes in CAD. Studies indicate that the nutritional status evaluated is linked to more extensive and complex coronary atherosclerosis in patients undergoing coronary angiography and is significantly associated with long-term cardiovascular outcomes in patients with stable CAD. Therefore, assessing nutritional status may be useful for the risk stratification of CAD patients [15,16,17].

There is a growing body of evidence linking visceral adiposity to long-term CVD risk and the severity of coronary atherosclerosis [18,19]. Yet, the evidence comparing visceral adiposity to general adiposity status in CAD is scarce. More importantly, the data on the association between visceral adiposity and nutritional status in CAD is lacking. Therefore, we set out to investigate visceral adiposity in relation to general adiposity and nutritional status in patients with stable CAD.

## 2. Materials and Methods

The study was approved by the bioethics board at the Medical University of Silesia and it conforms to the Declaration of Helsinki. The main inclusion criterion was stable CAD, which was defined as a history of documented myocardial infarction, prior coronary revascularization, chest pain with documented myocardial ischemia on noninvasive tests, or coronary stenosis of >50% proven by angiography. The exclusion criteria included: cancer (being under treatment and/or diagnosed with malignancies), chronic kidney disease—stage 4 or higher (baseline estimated glomerular filtration rate <60 mL/min/1.73 m^2^), uncontrolled thyroid dysfunction, liver dysfunction (including viral hepatitis, cholestatic jaundice with bilirubin concentration >1.5 mg/dL and/or alkaline phosphatase at least twice the upper limit of normal), coexisting autoimmune disorders, acute infectious disease, chronic inflammatory disease, uncontrolled diabetes mellitus, glucocorticoids and/or androgens therapy, the presence of signs or symptoms of fluid retention upon clinical examination at the index date (including orthopnea, ankle swelling, distended jugular veins, hepatomegaly, pulmonary rales, and the presence of pleural effusion), and lack of patient consent to participate. In addition, we excluded patients with cardiac implantable electronic devices (pacemakers, implantable cardioverter-defibrillators, cardiac resynchronizing therapy devices) and patients who had undergone bariatric surgery or procedures reducing gut absorptive capacity [20]. Consequently, we enrolled 204 patients with stable CAD hospitalized in the Department of Medicine and the Department of Geriatrics.

Based on the VAI-defined adipose tissue dysfunction (ATD) types, the study population (N = 204) was divided into four groups: (1) no ATD (N = 66), (2) mild ATD (N = 50), (3) moderate ATD (N = 48), and (4) severe ATD (N = 40) [14].

### 2.1. Anthropometric Measurements

We measured body weight to the nearest 0.05 kg with the use of a calibrated scale (B150L, Radwag, Radom, Poland). Height was measured to the nearest 0.1 cm with the use of a stadiometer (SECA 217). Hip circumference (HC) was measured at the level of the greater trochanter of the femoral bone. Waist circumference (WC) was measured at the smallest circumference between the costal margin and the iliac crest. Waist-to-hip ratio (WHR) was calculated as WC divided by HC. The waist-to-height ratio (WHtR) was calculated as WC divided by height.

Given that the median age in the entire study cohort was 70 years, we used age-specific cutoffs for the anthropometric indices.

BMI was calculated by dividing weight in kilograms by height in meters squared. Owing to criticism over the use of BMI in elderly populations due to the fact that it does not take into account age-associated changes, we used the National Research Council (US) Committee on Diet and Health classification of weight status in persons 65 years or older (Table 1) [21].

BAI was calculated according to the following formula [9]:

BAI=Hip circumference [cm](Height [m])^1.5−18

In the case of BAI, we used a sex- and age-specific classification of weight status (Table 1) [22].

Sex-specific formulae were used to calculate VAI [12].

$$\mathrm{Men}\;\mathrm{VAI}=\frac{\mathrm{WC}\;\left[\mathrm{cm}\right]}{39.68+\left(1.88\;\times \;\mathrm{BMI}\right)}\times \frac{\mathrm{TG}\;\left[\mathrm{mmol}/L\right]}{1.03}\times \frac{1.31}{\mathrm{HDL}\;\left[\mathrm{mmol}/L\right]}$$

$$\mathrm{Women}\;\mathrm{VAI}=\frac{\mathrm{WC}\;\left[\mathrm{cm}\right]}{36.58+\left(1.89\;\times \;\mathrm{BMI}\right)}\times \frac{\mathrm{TG}\;\left[\mathrm{mmol}/L\right]}{0.81}\times \frac{1.52}{\mathrm{HDL}\;\left[\mathrm{mmol}/L\right]}$$

In the case of VAI, we used an age-specific classification of adipose tissue dysfunction (ATD) (Table 1) [14].

### 2.2. Bioimpedance Analysis (BIA)

Body fat was evaluated via bioelectrical impedance analysis (BIA) using a Tanita instrument (TBF-300A). FM was estimated by BIA. BIA-derived body fat percentage (BF %) equations were used to estimate lean body mass (LBM), FFM, total body water (TBW) and body fat mass. We used the following conversions to estimate BF%: FFM = 0.97 × LBM for men and FFM = 0.92 × LBM for women; FFM = TBW/0.73; BF % = (body weight − FFM)/body weight [23,24].

### 2.3. Nutritional Status

Nutritional status was analyzed using the Controlling Nutritional Status (CONUT) score, which takes into account serum albumin levels (g/dL), serum total cholesterol level (mg/dL), and total lymphocyte count (/mm^3^) (Table 2) [25]. Thus, the CONUT score ranges from 0 to 12. Higher scores reflect worse nutritional status. Patients were classified as having normal nutritional status (0–1) or mildly (2–4), moderately (5–8), or severely (9–12) malnourished status based on the CONUT score.

### 2.4. Statistical Analysis

Quantitative data are presented as means and standard deviations or medians and interquartile ranges (lower and upper quartiles). Qualitative data are presented as frequencies. One-way analysis of variance (one-way ANOVA) or Kruskal–Wallis ANOVA were used to test the differences between the three groups. The relationship between VAI, BMI, and BAI were evaluated by Spearman’s rank correlation coefficient. A receiver operating characteristic (ROC) analysis was performed to assess the predictive value of identifying the discrimination thresholds of BAI and BMI for all VAI-defined ATDs. ROC curves for BMI and BAI were compared with the DeLong method. Multivariate logistic regression was used to evaluate the associations between adipose tissue dysfunction, BMI, and nutritional status. Four models were chosen to adjust for the differences in baseline characteristics. The results are given as odds ratios (ORs) and 95% confidence intervals (CI). In the case of continuous variables, ORs should be interpreted in terms of each unit increase on the scale. A value of *p* < 0.05 was considered significant.

## 3. Results

**Figure 1:**
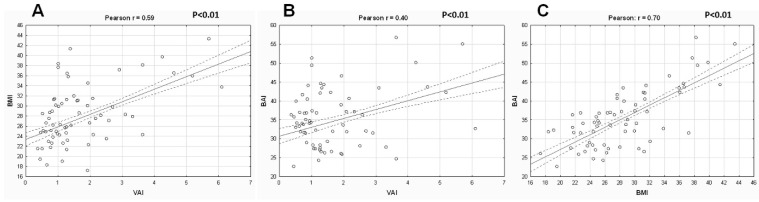
The correlation between (**A**) visceral adiposity index (VAI), (**B**) body adiposity index (BAI), and (**C**) body-mass index (BMI).

**Figure 2:**
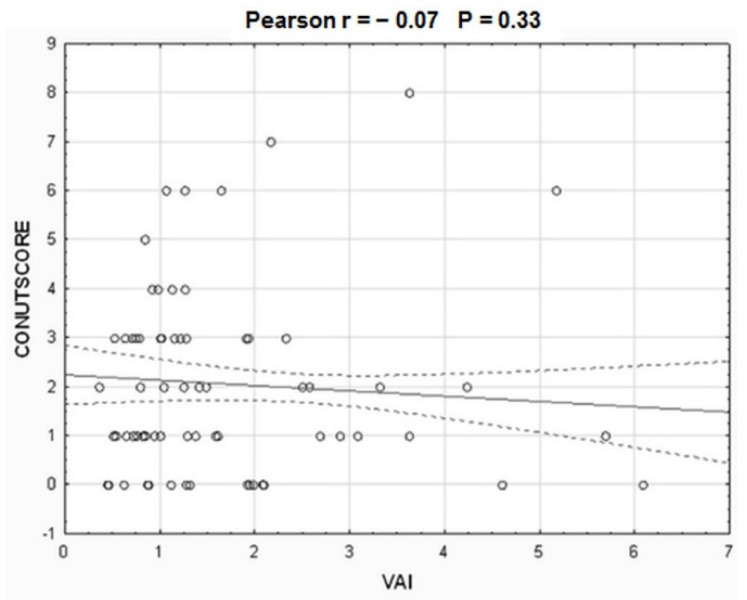
The correlation between visceral adiposity index (VAI) and body-mass index CONUT score.

Baseline clinical and laboratory characteristics are presented in Table 3. Patients with ATD were more commonly men. Patients with moderate or severe ATD were younger in comparison to patients with no or mild ATD. Hyperlipidemia was more prevalent in patients with moderate or severe ATD. This was also reflected in the lipid profile. Weight, BMI, BAI, and other anthropometric indices increased with the increasing severity of ATD across all four study groups (Table 4). The patients with severe ATD had twice the amount of fat in comparison to the patients with no ATD, which conferred a 1.5-fold increase in body fat percentage. Although the patients with severe ATD had the highest amount of total body water (median 36.3 kg), they had the lowest total body water percentage (median 39.2%) (*p* < 0.001) (Table 4). Patients with moderate and severe ATD were the youngest (median 67 years), yet their metabolic age was the eldest (median 80 and 84 years, respectively) in comparison to their counterparts with no or mild ATD (median 68 and 65 years, respectively) (Table 4). VAI had just a modest positive correlation with BAI and BMI, whereas BMI demonstrated a strong correlation with BAI (Figure 1). However, there was no correlation between VAI and CONUT scores (Figure 2). CONUT scores were similar across all four study groups. 

**Figure 3:**
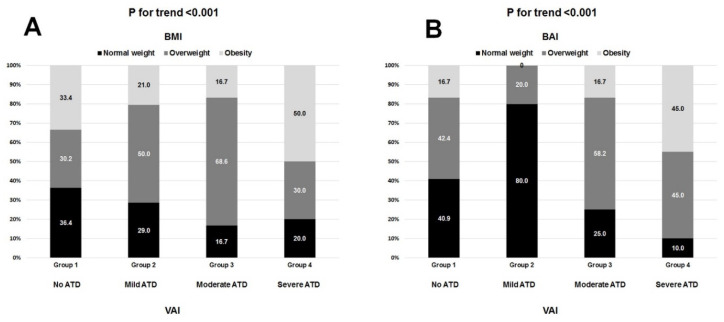
The distribution of BMI-defined (**A**) and BAI-defined (**B**) weight categories in respective adipose tissue dysfunction (ATD) types defined by VAI.

**Figure 4:**
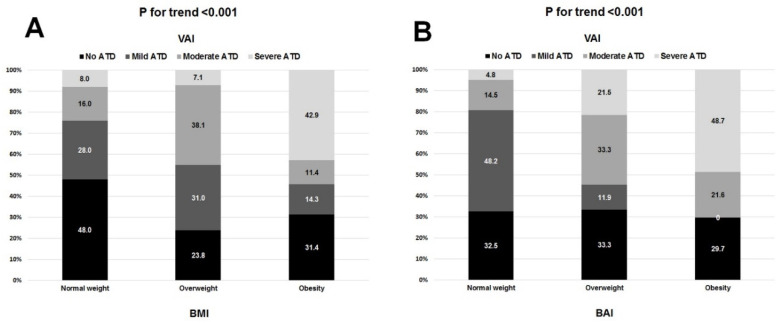
The distribution of VAI-defined adipose tissue dysfunction (ATD) types in respective BMI-defined (**A**) and BAI-defined (**B**) weight categories.

**Figure 5:**
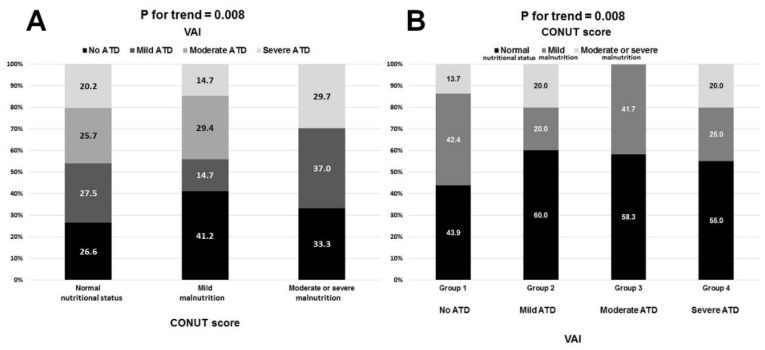
The distribution of VAI-defined adipose tissue dysfunction (ATD) types in respective CONUT score-defined nutritional status (**A**) and the distribution of nutritional status in respective VAI-defined types of adipose tissue dysfunction (ATD) (**B**).

**Figure 6:**
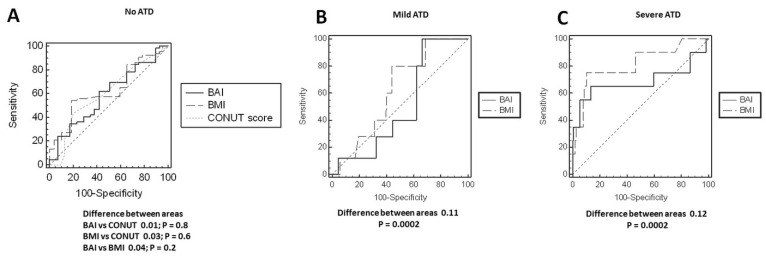
Difference between BMI and BAI receiver-operating characteristics (ROC) curves in identifying VAI-defined adipose tissue dysfunction (ATD): (**A**) no ATD, (**B**) Mild ATD, and (**C**) severe ATD.

Figure 3 depicts the high variability in the distribution of BMI-defined (Figure 3A) and BAI-defined (Figure 3B) weight categories across all four types of ATD. In particular, these discrepancies were seen in roughly 30–60% of participants with respect to BMI and 50–80% of participants with respect to BAI. Likewise, each of the BMI-defined and BAI-defined weight categories included patients with all forms of ATD (Figure 4A,B respectively). A total of 75% of patients with normal nutritional status had some form of ATD, and one-third of patients with moderate or severe malnutrition did not have any ATD (Figure 5A). By contrast, 55–60% of patients with mild, moderate, or severe ATD had normal nutritional status (Figure 5B). ROC analysis showed BMI, BAI, and CONUT scores to have a weak predictive value in determining no ATD (Table 5). Only BMI had a weak predictive value in determining mild ATD. Both the BMI and BAI had a strong predictive value for determining severe ATD, but the CONUT score did not have any discriminating power for mild, moderate, or severe ATD (Table 5). BAI, BMI, and CONUT score had similar predictive values for determining no ATD; however, BMI better predicted mild ATD and severe ATD than did BAI (difference between AUC 0.12 *p* = 0.0002) (Figure 6). BMI was associated with ATD in unadjusted and adjusted models, whereas nutritional status was not (Table 6). The risk of ATD increased by 9% per 1 kg/m^2^ increment in BMI in the unadjusted model (OR 1.09) and by 8–15% per 1 kg/m^2^ increment in BMI in four adjusted models (ORs 1.08, 1.11, 1.10, 1.15, respectively). 

In addition, BMI was associated with malnutrition in unadjusted and adjusted models (Table 6). The risk of malnutrition decreased by 11% per 1 kg/m^2^ increment in BMI in unadjusted model (OR 0.89) and decreased by 10–21% per 1 kg/m^2^ increment in BMI in four adjusted models (ORs 0.90, 0.88, 0.87, 0.79 respectively). Whereas VAI was only associated with malnutrition in the unadjusted model and after adjusting for sex and age (Table 6). The risk of malnutrition decreased by 21% per 0.5 unit increment of the VAI index (OR 0.79) and decreased by17% per 0.5 unit increment of the VAI index after adjusting the model for sex and age (OR 0.83).

## 4. Discussion

We have set out to investigate visceral adiposity in relation to body adiposity in patients with stable coronary artery disease. There are several key findings of this study. First and foremost, ATD was common in patients with CAD and accounted for two-thirds of the study population. Roughly one in five patients had severe ATD. Second, VAI demonstrated just a modest positive correlation with BAI and BMI, whereas BMI demonstrated a strong positive correlation with BAI. In addition, we identified a high variability in the distribution of BMI-defined and BAI-defined weight categories across all four types of ATD. Likewise, each of the BMI-defined and BAI-defined weight categories included patients with all forms of ATD. Finally, both the BMI and BAI had a strong predictive value for determining severe ATD. However, BMI predicted severe ATD better than the BAI did.

There is a large body of evidence linking obesity to CVD. Although the association between excess body weight and CAD risk is complex, abdominal obesity is believed to play a fundamental role via its adverse effect on several established CVD risk factors [1]. Several studies examined the link between BMI and CAD, but their results are conflicting. Labounty et al. investigated the association between BMI and the presence, extent, severity, and CAD risk among 13,784 patients referred for coronary computed tomographic angiography (CCTA) [26]. They reported that individuals with increased BMI had a greater prevalence, extent, and severity of CAD that could not be entirely explained by the presence of traditional risk factors. A higher BMI was independently related to an increased intermediate-term risk of myocardial infarction [26]. Similar results were reported by others. Flint et al. followed 27,859 men and 41,534 women for 16 years and found that both BMI and WC were strong predictors of the future risk of CAD [27]. By contrast, Niraj et al. demonstrated that obese patients who were referred for coronary angiography were younger and, thus, had a lower CAD burden, lending further credence to the notion that the “obesity paradox” does exist. However, after adjusting for other comorbid conditions, obesity in and of itself turned was not an independent predictor of the severity of CAD [28]. Similarly, Gregory et al. examined 8705 patients referred for coronary angiography and failed to demonstrate any association between BMI and 12-month all-cause or cardiac-specific mortality, even after adjusting for potential confounding variables [29].

In terms of body fat distribution, visceral adiposity plays a pivotal role in cardiometabolic risk and CVD outcomes [5]. Studies indicate that obese patients with metabolic abnormalities such as insulin resistance and atherogenic dyslipidemia were characterized by an excess of abdominal VAT, whereas obese patients who had a “normal” metabolic profile were characterized by subcutaneous accumulations of fat with low levels of VAT [30]. Notably, excess visceral adiposity may be indicative of a disrupted hormonal/inflammatory milieu affecting both regional fat distribution and cardiometabolic risk. Such a state is linked to a plethora of metabolic abnormalities, including insulin resistance, hyperinsulinemia, type 2 diabetes mellitus, atherogenic dyslipidemia, inflammatory state, altered cytokine profile, impaired fibrinolysis, elevated risk of thrombosis, and endothelial dysfunction [5]. Zhang et al. reported that the hypertriglyceridemic waist phenotype, together with a high VAI score (indicative of severe adipose tissue dysfunction), were linked to substantially elevated CAD risk in men and women [31]. A study of 24,508 men and women demonstrated that indices of abdominal obesity were more consistent and stronger predictors of CAD than was BMI [32]. In addition, several studies provided further evidence that CVD risk is more closely linked to body shape and adipose tissue distribution rather than to BMI or excess total body fat [33,34]. Notably, CVD patients with lower BMI values may nevertheless have high levels of visceral adipose tissue and ectopic fat, which could make them more vulnerable to adverse clinical outcomes and death. Accordingly, excess visceral adiposity/ectopic fat may be a key solution to the obesity paradox seen in cardiovascular medicine [35].

We found that roughly 50% of patients deemed normal weight by BMI demonstrated some extent of ATD. On the flip side, 30% of those categorized as obese by BMI had no ATD. Importantly, there are reports pointing to the discordance between BMI and BAI in assessing adiposity status [20,36]. Upon assessing adiposity status by BAI, we found that roughly 70% of those deemed normal weight by BMI demonstrated some form of ATD, whereas 30% of those deemed obese by BAI had no ATD. However, when speaking of assessing ATD, we demonstrated that only BMI had poor predictive value in determining mild ATD. By contrast, both BMI and BAI had strong predictive value for determining severe ATD. However, BMI predicted mild ATD and severe ATD better than BAI did. Although BAI is said to be a good tool to measure adiposity, it would seem that, in general, BAI does not overcome the limitations of BMI, in particular when it comes to assessing visceral adiposity [37].

Notably, when speaking of body adiposity/weight status, it is vital to consider nutritional status. Studies show that obese and malnourished patients have worse outcomes in comparison to their counterparts without malnutrition [38,39]. More importantly, nutritional status is linked to more extensive and complex coronary atherosclerosis in patients undergoing coronary angiography [15]. More importantly, there is evidence suggesting that nutritional status has a prognostic impact on CAD patients. Chen et al. analyzed a cohort of 3118 patients with CAD undergoing percutaneous coronary intervention (PCI) and found that after adjusting for comorbidities and medication, and increased CONUT score was independently associated with a higher risk of acute myocardial infarction (HR: 1.13; 95% CI: 1.03–1.24), cardiovascular death (HR: 1.18; 95% CI: 1.07–1.30), congestive heart failure (HR: 1.11; 95% CI: 1.04–1.18), a major adverse cardiovascular event (HR: 1.14; 95% CI: 1.07–1.22), and total cardiovascular events (HR: 1.11; 95% CI: 1.07–1.15) [17]. To the best of our knowledge, this is the first study investigating the relationship between visceral adiposity and nutritional status. We have demonstrated that the majority of patients with normal nutritional status had some form of ATD and as much as one-third of patients with moderate or severe malnutrition did not have any ATD. By contrast, roughly half of the patients with mild, moderate, or severe ATD had normal nutritional status. Notwithstanding, there is some evidence published linking malnutrition to the development of visceral adiposity. Recent studies have pointed out that malnutrition prior to birth plays a vital role in triggering predisposition to visceral obesity and metabolic disorders [40].

Another significant aspect of the discordance in determining visceral adiposity and ATD is the ambiguities surrounding the appropriate level of visceral obesity that should trigger an intervention due to the lack of consensus on specific criteria and cutoffs. This fact remains an important barrier when assessing patients on an individual level, especially with regard to implementing weight reduction strategies. The alternatives to BMI deserve serious consideration in light of their implications for health, preventative or otherwise. In particular, VAI—which determines visceral adipose tissue dysfunction—could be an additional tool facilitating the timing of the implementation of appropriate non-pharmacological and pharmacological interventions in patients with CAD.

Let us now consider the aspect of nutritional status in the elderly. Diet and nutritional status are crucial determinants of health and disease among the elderly. The complex interplay between dietary, socioeconomic, physical, and psychological features contribute to nutritional status in older patients [41]. Malnutrition in the elderly features insufficient dietary intake, poor appetite, muscle wasting, and weight loss, which result in adverse effects on physiological functions and other clinical outcomes, including worse quality of life, susceptibility to infections, muscle wasting, increased risk of hospitalization, and rehospitalization, or even poor prognosis [42]. Therefore, malnutrition in the elderly in terms of body composition and visceral ATD should be considered for an accurate definition of the standards for the intensity of clinical interventions (nutritional, pharmacological, psychological, rehabilitation, and surgical).

## 5. Conclusions

In summary, this study has shown a high rate of misclassification of visceral ATD by BMI and BAI. Notably, ATD and malnutrition were common in patients with CAD. Roughly one in five patients had severe ATD. We identified a high level of variability in the distribution of BMI-defined and BAI-defined weight categories across all four types of ATD. Likewise, each of the BMI-defined and BAI-defined weight categories included patients with all forms of ATD. In addition, the majority of patients with normal nutritional status had some form of ATD, and as many as one-third of patients with moderate or severe malnutrition did not have any ATD.

These findings have important clinical implications for everyday practice because the line between health and disease in malnutrition in terms of body composition and visceral ATD is a significant consideration for an accurate definition of the standards for the intensity of clinical interventions (nutritional, pharmacological, psychological, rehabilitation, and surgical).

## 6. Study Limitations

One source of weakness in this study was the relatively small number of patients that could have rendered some differences insignificant. Thus, further studies need to be carried out in order to validate our results, especially in younger populations. The validity of the VAI was not evaluated in the Polish population. Likewise, further experiments using DXA could shed more light on visceral adiposity in relation to body adiposity.

The study participants were largely elderly (mean age was 70 years). Thus, the results of the study cannot be generalized. Similar studies are warranted in younger populations. Notwithstanding, given the aging of the population (especially this with cardiovascular disease) and the importance of an increased nutritional risk among the elderly, the results of the current study should be viewed as relevant in clinical practice.

## Figures and Tables

**Table 1:** Age- and sex-specific cutoffs for assessing visceral adiposity and body adiposity via the visceral adiposity index (VAI), body-mass index (BMI), and body adiposity index (BAI).

**VAI**		
Visceral Adiposity	Age ≥ 66 years	
No ATD	≤2.00	
Mild ATD	2.01–2.41	
Moderate ATD	2.42–3.17	
Severe ATD	>3.17	
**BMI**		
Weight status	Age ≥ 65 years	
Underweight	≤24.0	
Normal weight	24.1–29.0	
Overweight	29.1–35.0	
Obesity	>35.0	
**BAI**		
Weight status	Age ≥ 60 years	
	Men	Women
Underweight	<13%	<25%
Normal weight	13–25%	25–38%
Overweight	26–31%	39–43%
Obesity	>31%	>43%

ATD—adipose tissue dysfunction; BAI—body adiposity index; BMI—body-mass index; VAI—visceral adiposity index.

**Table 2:** The Controlling Nutritional Status (CONUT) score.

Parameter	Score			
Serum albumin [g/mL]	≥3.50	3.00–3.49	2.50–2.99	<2.50
Albumin score	0	2	4	6
Total cholesterol [mg/dL]	>180	140–180	100–139	<100
Cholesterol score	0	1	2	3
Lymphocyte count [count/mL]	≥1600	1200–1599	8000–1199	<800
Lymphocyte score	0	1	2	3
Total CONUT score	0–1	2–4	5–8	9–12
Nutritional status	Normal	Mild malnutrition	Moderate malnutrition	Severe malnutrition

**Table 3:** Baseline clinical and laboratory characteristics.

	Group 1 No ATD (N = 66)	Group 2 Mild ATD (N = 50)	Group 3 Moderate ATD (N = 48)	Group4 Severe ATD (N = 40)	*p*
Sex, men n (%)	22 (34.8)	16 (32.0)	20 (41.7)	18 (45.0)	<0.001
Age (years)	73 ± 5	72 ± 7	67 ± 3	67 ± 3	<0.001
Prior myocardial infarction n (%)	16 (24.0)	15 (30.0)	12 (25.0)	14 (35.0)	0.40
Heart failure n (%)	31 (47.0)	24 (48.0)	24 (50.0)	18 (45.0)	0.97
Atrial fibrillation n (%)	9 (13.6)	0 (0)	12 (25.0)	10 (25.0)	0.01
Hyperlipidemia n (%)	20 (33.3)	15 (30.0)	20 (41.7)	20 (50.0)	0.02
Diabetes mellitus n (%)	23 (24.8)	18 (36.0)	12 (25.0)	16 (40.0)	0.47
Hypertension n (%)	59 (89.4)	48 (96.0)	46 (96.0)	28 (70.0)	<0.001
CONUT score	2 (1–3)	1 (0–3)	1 (1–2)	1 (0–2)	0.18
Nutritional status					
Normal	29 (43.9)	30 (60.0)	28 (58.3)	22 (55.0)	
Mild malnutrition	28 (42.4)	10 (20.0)	20 (41.7)	10 (25.0)	0.008
Moderate or severe malnutrition	9 (13.7)	10 (20.0)	0 (0)	8 (20.0)	
Leucocytes (10^3^/mm^3^)	6.9 ± 2.9	7.9 ± 3.2	7.8 ± 1.6	7.9 ± 2.0	0.10
Erythrocytes (10^6^/mm^3^)	4.2 ± 0.5	4.3 ± 0.3	4.3 ± 0.5	4.3 ± 0.5	0.14
Hemoglobin (g/dL)	12.3 ± 1.5	12.8 ± 0.6	12.3 ± 1.2	13.4 ± 1.5	0.01
Hematocrit (%)	38 ± 4	39 ± 2	38 ± 3	41 ± 4	0.01
Platelets (10^3^/mm^3^)	243 ± 70	197 ± 24	263 ± 89	189 ± 34	<0.001
Total cholesterol (mmol/L)	4.3 ± 1.0	5.2 ± 1.9	4.6 ± 0.5	4.6 ± 1.1	<0.001
HDL cholesterol (mmol/L)	1.5 ± 0.4	1.2 ± 0.2	0.9 ± 0.2	0.7 ± 0.1	<0.001
LDL cholesterol (mmol/L)	2.3 ± 0.9	3.3 ± 1.8	2.7 ± 0.8	2.9 ± 0.8	<0.001
Triglycerides (mmol/L)	1.1 ± 0.3	1.8 ± 0.4	1.7 ± 0.4	2.4 ± 0.7	<0.001
Serum creatinine (μmol/L)	80 ± 25	73 ± 14	95 ± 32	91 ± 38	<0.001
eGFR (mL/min/1.73 m^2^)	77 ± 20	79 ± 26	69 ± 25	75 ± 22	0.14

eGFR—estimated glomerular filtration rate; HDL—high-density lipoprotein; LDL—low-density lipoprotein.

**Table 4:** Anthropometric and body composition measurements.

	Group 1 No ATD (n = 66)	Group 2 Mild ATD (n = 50)	Group 3 Moderate ATD (n = 48)	Group4 Severe ATD (n = 40)	*p*
Weight (kg)	72 (59–79)	63 (62–65)	70 (64–77)	89 (87–95)	<0.001
Height (m)	1.62 (1.51–1.70)	1.64 (1.50–1.71)	1.65 (1.55–1.73)	1.65 (1.55–1.72)	0.7
BMI	26 (24–31)	27 (24–28)	28 (27–30)	36 (3039)	<0.001
VAI	1.05 (0.80–1.37)	2.09 (2.00–2.17)	2.69 (2.57–2.99)	4.60 (3.62–5.43)	<0.001
BAI	34.1 (28.4–40.0)	36.2 (32.0–37.2)	34.2 (31.6–39.9)	43.7 (30.3–52.6)	<0.001
Hip circumference (cm)	104 (94–110)	102 (101–105)	109 (102–112)	120 (110–125)	<0.001
Waist circumference (cm)	103 (87–109)	100 (93–111)	113 (110–118)	122 (111–126)	<0.001
Mid-upper arm circumference (cm)	28 (25–31)	29 (28–32)	27 (26–29)	30 (27–32)	0.7
Calf circumference (cm)	34 (33–36)	32 (31–35)	33 (30–37)	39 (38–40)	<0.001
Waist-to-hip ratio	0.97 (0.90–1.00)	1.00 (0.95–1.03)	1.04 (1.00–1.07)	1.00 (0.97–1.02)	<0.001
Waist-to-height ratio	0.61 (0.57–0.69)	0.65 (0.56–0.74)	0.69 (0.68–0.76)	0.77 (0.67–0.83)	<0.001
Fat (%)	31.6 (25.0–39.6)	37.8 (33.4–42.2)	43.3 (26.6–45.0)	44.6 (33.7–46.7)	<0.001
Fat (kg)	20.7 (15.2–31.6)	23.5 (21.1–26.8)	28.6 (20.6–31.6)	41.2 (26.9–43.7)	<0.001
Visceral fat rating	13 (10–16)	13 (10–14)	15 (14–18)	16 (15–19)	<0.001
Lean mass (kg)	42.9 (40.0–55.5)	38.9 (37.8–42.2)	42.5 (36.8–56.9)	50.6 (48.2–55.3)	<0.001
Total body water (%)	47.3 (41.3–51.1)	42.1 (39.2–45.8)	41.0 (37.1–49.6)	39.2 (37.9–45.5)	<0.001
Total body water (kg)	29.7 (27.0–37.6)	26.4 (26.2–29.0)	28.7 (24.8–40.1)	36.3 (34.1–39.5)	<0.001
Metabolic age	68 (65–77)	65 (60–71)	80 (68–83)	84 (74–88)	<0.001

ATD—adipose tissue dysfunction; BAI—body adiposity index; BRI—body roundness index; BMI—body-mass index; VAI—visceral adiposity index.

**Table 5:** Receiver-operating characteristics (ROC) curves identifying discrimination thresholds of BAI and BMI for VAI-defined adipose tissue dysfunction (ATD).

**No ATD**							
	**cutoff**	**AUC (95%CI)**	**Sensitivity (%)**	**Specificity (%)**	**PPV (%)**	**NPV (%)**	***p***
BMI	<26	0.63 (0.56–0.69)	55	81	58	79	0.003
BAI	<35.9	0.59 (0.52–0.66)	62	58	41	76	0.04
CONUT score	<2	0.60 (0.53–0.67)	44	80	51	75	0.01
**Mild ATD**							
	**cutoff**	**AUC (95%CI)**	**Sensitivity (%)**	**Specificity (%)**	**PPV (%)**	**NPV (%)**	***p***
BMI	<28	0.62 (0.55–0.69)	80	56	37	89	0.003
BAI	<40.8	0.51 (0.44–0.58)					0.8
CONUT score	0	0.60 (0.48–0.65)					0.1
**Moderate ATD**							
	**cutoff**	**AUC (95%CI)**	**Sensitivity (%)**	**Specificity (%)**	**PPV (%)**	**NPV (%)**	***p***
BMI	<30	0.49 (0.42–0.56)					0.9
BAI	<36.3	0.55 (0.48–0.62)					0.2
CONUT score	>2	0.51 (0.44–0.58)					0.8
**Severe ATD**							
	**cutoff**	**AUC (95%CI)**	**Sensitivity (%)**	**Specificity (%)**	**PPV (%)**	**NPV (%)**	**P**
BMI	>32	0.78 (0.68–0.87)	75	90	64	94	<0.0001
BAI	>42.3	0.66 (0.62–0.75)	65	86	54	91	0.003
CONUT score	<5	0.48 (0.41–0.55)					0.7

AUC—area under the curve; BAI—body adiposity index; BMI—body-mass index; CI—confidence interval; NPV—negative predictive value; PPV—positive predictive value; ROC—receiver-operating characteristics.

**Table 6:** Multivariate logistic regression models to evaluate the associations between adipose tissue dysfunction, BMI, and nutritional status.

Variable	OR	95%CI	*p*
**Adipose tissue dysfunction (mild, moderate, or severe)**			
BMI (unadjusted)	1.09	1.03–1.16	0.003
BMI (adjusted—Model 1)	1.08	1.01–1.15	0.02
BMI (adjusted—Model 2)	1.11	1.03–1.18	0.003
BMI (adjusted—Model 3)	1.10	1.03–1.18	0.005
BMI (adjusted—Model 4)	1.15	1.06–1.24	<0.0001
In the case of BMI, OR should be interpreted in terms of one unit increase on the scale [per 1 kg/m^2^ increment]. Model 1—adjusted for age, sex, Model 2—adjusted for age, sex, hyperlipidemia, hypertension, Model 3—adjusted for age, sex, hyperlipidemia, hypertension, nutritional status, Model 4—adjusted for age, sex, hyperlipidemia, hypertension, nutritional status, total cholesterol			
CONUT score (unadjusted)	0.91	0.80–1.05	0.16
CONUT score (adjusted—Model 1)	0.90	0.83–1.07	0.30
CONUT score (adjusted—Model 2)	0.92	0.80–1.05	0.25
CONUT score (adjusted—Model 3)	0.97	0.84–1.13	0.70
CONUT score (adjusted—Model 4)	1.28	0.91–1.68	0.21
In the case of CONUT score, OR should be interpreted in terms of a one point increase in the score [per 1 point increment]. Model 1—adjusted for age, sex, Model 2—adjusted for age, sex, hyperlipidemia, hypertension, Model 3—adjusted for age, sex, hyperlipidemia, hypertension, BMI, Model 4—adjusted for age, sex, hyperlipidemia, hypertension, BMI, total cholesterol			
**Malnutrition (mild, moderate, or severe)**			
BMI (unadjusted)	0.89	0.83–0.94	<0.0001
BMI (adjusted—Model 1)	0.90	0.84–0.95	<0.0001
BMI (adjusted—Model 2)	0.88	0.83–0.94	<0.0001
BMI (adjusted—Model 3)	0.87	0.81–0.93	<0.0001
BMI (adjusted—Model 4)	0.79	0.72–0.87	<0.0001
In the case of BMI, OR should be interpreted in terms of one unit increase on the scale [per 1 kg/m^2^ increment]. Model 1—adjusted for age, sex, Model 2—adjusted for age, sex, hyperlipidemia, hypertension, Model 3—adjusted for age, sex, hyperlipidemia, hypertension, VAI, Model 4—adjusted for age, sex, hyperlipidemia, hypertension, VAI, total cholesterol			
VAI (unadjusted)	0.79	0.65–0.98	0.03
VAI (adjusted—Model 1)	0.83	0.67–0.99	0.04
VAI (adjusted—Model 2)	0.88	0.69–1.12	0.30
VAI (adjusted—Model 3)	1.22	0.90–1.67	0.19
VAI (adjusted—Model 4)	1.33	0.85–1.73	0.22
In the case of VAI, OR should be interpreted in terms of 0.5 unit increase of the index [per 0.5 unit increment of the index]. Model 1—adjusted for age, sex, Model 2—adjusted for age, sex, hyperlipidemia, hypertension, Model 3—adjusted for age, sex, hyperlipidemia, hypertension, BMI, Model 4—adjusted for age, sex, hyperlipidemia, hypertension, BMI, total cholesterol			

## Data Availability

The data presented in this study are available on request from the corresponding author. The data are not publicly available due to restrictions that apply to the availability of these data.
